# Integrating Physiological, Genetic, and Agronomic Approaches for Increasing Wheat Productivity: An Editorial Commentary

**DOI:** 10.3390/plants15040568

**Published:** 2026-02-11

**Authors:** Valentina Spanic, Milan Mirosavljević

**Affiliations:** 1 Department for Breeding & Genetics of Small Cereal Crops, Agricultural Institute Osijek, Juzno Predgradje 17, 31000 Osijek, Croatia; 2 Small Grains Department, Institute of Field and Vegetable Crops Serbia, Maksima Gorkog 30, 21000 Novi Sad, Serbia

Wheat (*Triticum aestivum* L.) is one of the most important cereal crops worldwide, serving as a primary source of calories, protein, and essential nutrients for a large proportion of the global population. As the demand for food continues to rise due to population growth, climate change, and shrinking arable land area, increasing the agronomic performance and nutritional quality of wheat have become critical priorities for ensuring sustainable agriculture and global food security. Agronomic traits such as grain yield, drought and heat tolerance, disease resistance, and adaptability to diverse agro-ecological conditions directly influence wheat productivity and stability. Traditional breeding approaches have significantly contributed to grain yield increases; however, yield gains have slowed due to the effects of biotic and abiotic stresses, including pests, pathogens, salinity, and extreme weather events. Therefore, enhancing agronomic traits through modern breeding strategies, including the use of marker-assisted selection, genomic selection, and biotechnological tools, is essential for ensuring consistent wheat production under changing environmental conditions.

The Special Issue of *Plants* titled “Improvement of Agronomic Traits and Nutritional Quality of Wheat” includes five articles and one review that address the global challenges facing wheat production by improving agronomic performance and grain quality. This collection gathers studies on the physiological traits associated with disease resistance and grain yield stability, the influences of climate and environment on wheat breeding sites, and the genome-wide identification of resistance loci in wheat. The contributions underscore the need for integrated approaches combining traditional breeding with modern genomic and physiological tools for developing high-yielding, stress-resilient, and nutritionally improved wheat varieties that meet the demands of sustainable food systems under changing environments.

One study investigated the effects of elevated atmospheric CO_2_ concentrations on carbohydrate utilization in wheat cultivars with contrasting ear architectures, finding that large-ear wheat cultivars benefit more from elevated CO_2_ levels through enhanced non-structural carbohydrate accumulation, efficient carbohydrate remobilization to grains, and sustained photosynthetic activity during grain filling [1]. These physiological advantages resulted in larger kernel weight and stable grain yield across years, highlighting ear architecture as a key trait for breeding wheat cultivars adapted to future environments with elevated CO_2_ levels. Overall, these findings underscore the central role of wheat ear architecture as a major determinant of source–sink balance, carbon utilization efficiency, and grain yield stability, particularly under elevated CO_2_ level conditions, highlighting wheat ears as a key target trait for breeding climate-resilient, high-yielding cultivars [1].

Biotic stress resistance is addressed in the second article in this Special Issue. Spanic et al. [2] demonstrated that dynamic leaf physiology and architectural traits play a significant role in shaping resistance to Fusarium head blight (FHB), a major disease affecting wheat yield and grain quality. In a field experiment, they measured photosynthetic activity, water relationships, and leaf angular traits in FHB-resistant and FHB-susceptible genotypes at 10 and 18 days after inoculation. The FHB-resistant genotypes exhibited the early and coordinated regulation of stomatal conductance, transpiration, leaf water status, photosynthetic efficiency, and leaf orientation. FHB-susceptible genotypes showed disrupted physiological responses. These findings indicate that early physiological and morphological responses may serve as phenotypic markers for rapid screening and breeding of FHB-resistant wheat varieties, linking functional traits to improved grain yield stability and grain quality under disease pressure. This study underscores the importance of integrating physiological and morphological approaches in breeding strategies for developing wheat cultivars that are high-performing and resilient to biotic stresses.

Environmental adaptability and yield stability were explored by Morgounov et al. [3] in the third article in this Special Issue. They evaluated the climate, weather, and ecological factors influencing high-latitude spring wheat breeding sites and germplasm performance. Their study highlights the importance of genotype (G)–environment (E) interactions in shaping agronomic traits under challenging growing conditions, demonstrating that regional climate trends, including rising temperatures and variable precipitation, can significantly affect spring wheat’s yield potential. Other studies of wheat production have similarly emphasized the importance of integrating climate data and G×E analysis to guide breeding for yield stability under changing environmental conditions. These findings underscore the need for breeding programs that integrate environmental and ecological data to develop adapted, high-yielding wheat cultivars capable of maintaining productivity in high-latitude environments.

The fourth article, a review, is dedicated to preharvest sprouting (PHS), the premature germination of mature wheat kernels that reduces grain yield and quality and is influenced by genetic and environmental factors. Khumalo-Mthembu et al. [4] reviewed breeding efforts in South Africa, highlighting that conventional breeding has led to the development cultivars with durable PHS tolerance, but progress has been limited by the trait’s polygenic nature, germplasm constraints, and slow adoption of modern genomic tools. This review highlights genetic and environmental factors affecting sprouting resistance and discussed current breeding progress and future prospects, linking agronomic performance with grain quality preservation.

Disease resistance in wheat was further addressed by Ma et al. [5] in the fifth article in this Special Issue. Ma et al. conducted a genome-wide association study (GWAS) to identify the genetic resources conferring resistance to stripe rust (*Puccinia striiformis* f. sp. *tritici*). Screening 198 modern wheat varieties with prevalent *Pst* races in the seedling and adult plant stages, they identified seven elite cultivars with stable high-level resistance and 14 significant quantitative trait loci, including nine potentially novel loci. Candidate genes and favorable haplotypes were characterized, providing molecular tools for pyramiding resistance genes. These findings demonstrate the value of integrating phenotyping, genomic analysis, and haplotype information to accelerate the breeding of durable, stripe-rust-resistant wheat varieties.

Agronomic management practices were addressed by Mirosavljević et al. [6], who analyzed yield determination in response to nitrogen fertilization in small grain crops, providing insights into management practices for optimizing nutrients to improve productivity. Small grain crops show significant variation in grain yield (GY) and its components under different nitrogen (N) fertilization levels. This study evaluated triticale, wheat, six-rowed, and two-rowed barley across multiple sites and seasons, focusing on grain number (GN), grain weight (GW), spike number (SN), spike dry weight (SDWa), and fruiting efficiency (FE). Triticale had the highest GY, driven by higher GN and SDWa, while wheat exhibited the highest FE, reflecting efficient assimilate use. Two-rowed barley had high SN and SDWa but lower GN and FE, limiting yield. Nitrogen improved the yield components across all crops, though trade-offs between GW and other traits were observed, highlighting the importance of tailored fertilization and the further exploration of the genetic and physiological mechanisms underlying yield determination.

Overall, the articles and review published in this Special Issue demonstrate that improving wheat’s agronomic traits and nutritional quality require a multidisciplinary approach combining physiological studies, genomic analyses, breeding strategies, and optimized agronomic management. The findings contribute valuable knowledge for developing high-yielding, stress-resilient, and higher-quality wheat cultivars suitable for sustainable production under current and future environmental conditions.
